# The Effect of Language Barrier and Non-professional Interpreters on the Accuracy of Patient-physician Communication in Emergency Department

**DOI:** 10.22114/ajem.v0i0.123

**Published:** 2019-06-02

**Authors:** Ali Labaf, Amir Shahvaraninasab, Hamidreza Baradaran, Javad Seyedhosseini, Amirhosein Jahanshir

**Affiliations:** 1. Department of Emergency Medicine, Imam Khomeini Hospital Complex, Tehran University of Medical Sciences, Tehran, Iran.; 2. Prehospital and Hospital Emergency Research Center, Tehran University of Medical Sciences, Tehran, Iran.; 3. Department of Emergency Medicine, Imam Reza Hospital, AJA University of Medical Sciences, Tehran, Iran.; 4. Endocrine Research Center, Institute of Endocrinology and Metabolism, Iran University of Medical Sciences, Tehran, Iran.; 5. Department of Emergency Medicine, Shariati Hospital, Tehran University of Medical Sciences, Tehran, Iran.

**Keywords:** Communication Barriers, Communication Disorders, Cornell Medical Index, Emergency Medicine

## Abstract

**Introduction:**

Patients’ relatives commonly play the role of interpreters in medical interviews. These non-professional interpreters are prone to potentially-dangerous translation errors.

**Objective:**

The present study was conducted to evaluate these errors in the emergency department (ED).

**Method:**

Twenty interviews with Azeri patients were recorded. They were unable of speaking Persian and therefore accompanied by a relative as a Persian interpreter. These records were presented to two physicians as native Azeri speakers to determine the clinical importance of the interpreters’ errors according to their medical expertise.

**Results:**

The total omission and addition errors observed in Azeri to Persian translation were significantly more than in Persian to Azeri translation, while mistranslation errors were almost the same. The relatives with higher levels of education made fewer errors, and those living with the patients made significantly more addition errors.

**Conclusion:**

Non-professional interpreters cannot effectively facilitate patient-physician communication, as their translation is error-prone, especially in terms of translating their native language into official languages. These errors can have important clinical ramifications.

## Introduction

Patient-physician communication is the cornerstone of effective medical interviews that is an important task that is performed by physicians regularly in emergency department (ED) (1, 2). Communication with patients has been defined as “specific tasks and observable behaviors including conducting interviews to obtain a medical history and clarify patient reason for the visit, discussing a diagnosis and prognosis, giving therapeutic instructions and the information required for patient informed consent before their undergoing any medical procedures as well as counselling to encourage participation in treatments to relieve symptoms” (3). Although physicians should be aware of non-verbal clues in patient behavior, verbal communication still plays a leading role in patient-physician communication. Effectively using a language can strengthen the patient-physician relationship, and result in a better communication; nevertheless, there are some obstacles such as inappropriate use of language and language barrier in this regard (4). Using interpreters is the common solution for language barrier. Although using professional interpreters is the standard recommendation, many patients use their bilingual relatives as interpreters, since professional interpreters are not available (5, 6). These non-professional interpreters have been shown to make certain interpretation errors, which potentially compromises patient safety (7). The present research was performed to evaluate these errors and their clinical significance in the ED, which is more complex than outpatient clinics and wards.

## Methods

### Study design

The present study was conducted during 2016 on Azeri patients presenting to the EDs of two referral hospitals affiliated to Tehran University of Medical Sciences (TUMS). Ethical aspects of the current study were approved by ethics committee of TUMS, and patients entered the study if they signed the informed consent.

### Study population

The inclusion criteria comprised inability to speak Persian and being accompanied by a bilingual relative as the interpreter. The exclusion criteria consisted of aphasia, decreased levels of consciousness, altered mental status, resuscitation requirements, being unwilling or unable to participate in the study and belonging to Azeri-speaking patients from the Republic of Azerbaijan. The authors were aware of the difficulties lying in finding Azeri patients who met the inclusion criteria given that the official language in Iran is Persian, and almost all young people speak Persian well, though accented. Sampling of all the eligible patients was therefore conducted in a six-month period.

### Data gathering

A member of the research team, who was unfamiliar with the Azeri language, interviewed all the eligible patients after obtaining their and their relatives’ verbal consent. The interviews were focused on a formal history taking session, and comprised questions on the patients’ chief complaints, present illnesses and their medical, familial and socio-economic histories and the other relevant factors. The patients’ relatives overcame the language obstacle by interpreting the conversations from Persian to Azeri and Azeri to Persian. They were asked to translate all of the physician-patient conversation without omitting or adding anything. Each session lasted about 15 minutes, and was recorded on a handheld smartphone for analysis. Socio-demographic information of the patients and their relatives were documented at the end of the interviews. The mp3 file of the recorded interviews was presented to two reviewers as native Azeri- speaking physicians. They were both asked to listen to the interviews and identify the interpreters’ errors in Persian to Azeri and Azeri to Persian translations in terms of omission, addition and mistranslation. They were also asked to identify the errors with significant effects on diagnosis and treatment based on their medical expertise.

### Definitions

The errors were categorized in three groups defined as follows:

- Omission: The interpreter’s omission of a word or phrase from what is expressed by the patient/physician
- Addition: The interpreter’s addition of a word or phrase to what is expressed by the patient/physician
- Mistranslation: The interpreter’s use of a wrong word or phrase for what is expressed by the patient/physician.

### Statistical analysis

Descriptive values were reported as frequency and percent. Inter-rater reliability of the two reviewers were measured by Kappa Coefficient test.

## Results

The six out of 26 eligible patients excluded consisted of two patients from the Republic of Azerbaijan, two who were unwilling to participate in the study and two who required resuscitation. Finally, 20 patients with the mean age of 64.70 years were enrolled, of whom 11 cases (55%) were female. Table 1 presents the sociodemographic information of the participants, and table 2 the results of the reviewers’ reports and inter-rater reliability. The reviewers reported many translation errors with unanimous agreement on omission and addition based on the kappa coefficient, although they fairly agreed on mistranslation errors. The total frequency of omission and addition errors in Azeri to Persian translation was significantly higher than that of Persian to Azeri translation, while mistranslation errors were almost equal in number.

**Table 1:** The sociodemographic information of the participants

**Variable**	
**Gender**	Frequency (%)
Male	9 (45.0)
Female	11 (55.0)
**Marital status**	
Single	0 (0.0)
Married	7 (35.0)
Divorced/Widowed	13 (65.0)
**Relatives’ education level**	
< 12 years	7 (35.0)
12–16 years	9 (45.0)
>16 years	4 (20.0)
**Patients’ education level**	
Illiterate	20 (100.0)
1–12 years	0 (0.0)
>12 years	0 (0.0)
**The patients and their relatives living together**	
Yes	15 (75.0)
No	5 (25.0)

**Table 2:** The results of the reviewers’ reports

**Error**	**Reviewer 1**			**Reviewer 2**			**Inter-rater Reliability**
	**Significant**	**Insignificant**	**Total**	**Significant**	**Insignificant**	**Total**	**Kappa Coefficient**
	**Frequency (%)**			**Frequency (%)**			
**Persian to Azeri**							
Omission	0 (0.0)	1 (100.0)	1	0 (0.0)	1 (100.0)	1	1.00
Addition	3 (42.9)	4 (57.1)	7	3 (42.9)	4 (57.1)	7	1.00
Mistranslation	2 (66.7)	1 (33.3)	3	2 (33.3)	4 (66.7)	6	0.58
**Azeri to Persian**							
Omission	5 (38.5)	8 (61.5)	13	6 (46.2)	7 (53.8)	13	1.00
Addition	10 (29.4)	24 (70.6)	34	10 (29.4)	24 (70.6)	34	0.82
Mistranslation	2 (66.7)	1 (33.3)	3	2 (40.0)	3 (60.0)	5	0.69

Table 3 classifies the frequency of errors made by the relatives by their education level and whether they were living with the patients. The differences were only significant in terms of addition errors.

Moreover, the group with an education level of over 16 years made fewer errors in Persian to Azeri translation compared to the group of 12–16 years, and made the fewest errors in Azeri to Persian translation compared to the other two groups. The relatives living with the patients also made significantly more addition errors in both Azeri to Persian and Persian to Azeri translations.

**Table 3:** The percentage of the relatives making translation errors by their education level and living status

**Error**	**Education Level**			**The patients and their relatives living together**	
	**< 12 years (Percentage)**	**12–16 years (Percentage)**	**>16 years (Percentage)**	**Yes (Percentage)**	**No (Percentage)**
**Persian to Azeri**					
Omission	14.3	0	0	6.7	0
Addition	28.6	55.6	0	46.7	0
Mistranslation	21.4	11.1	50.0	16.7	40.0
**Azeri to Persian**					
Omission	57.1	44.4	25.0	40.0	60.0
Addition	100.0	88.9	37.5	100.0	30.0
Mistranslation	21.4	27.8	0	20.0	20.0

## Discussion

According to the census conducted by the Statistical Centre of Iran (8), the mean age of the study patients was more than that of the general public, which sounds reasonable, as non-traumatic patients below the age of 14 years were not admitted to the hospital. The inclusion and exclusion criteria have also affected the mean age of the subjects, as almost all younger people, who were familiar with Persian, were excluded. Given that public education is officially based on Persian in all Iranian provinces, the majority of the study sample were expected to be illiterate. The higher prevalence of illiteracy in older ages also increased the mean age of the sample. Given the significantly higher frequency of errors in Azeri to Persian translation compared to in Persian to Azeri translation, the interpreters are expected to have had difficulties in either understanding Azeri or speaking Persian. Hammer et al. showed that using native language does not adversely affect the vocabulary acquisition of a second language. The interpreters appear not to have any problems with speaking Persian given that all were educated and that the official language in Iranian schools and universities is Persian. They may therefore lack the opportunity to use Azeri in daily communication, and have a limited Azeri vocabulary. This is the main problem in using a nonprofessional bilingual interpreter, as getting acquainted with a language as a result of its daily usage can negatively affect the vocabulary of the other language even if it is native. Sentences can be translated by interpreters using simultaneous or consecutive approaches each with their own disadvantages (9). According to the simultaneous approach, interpreters generally use a “word for word” method, which causes mistranslation errors and lowers inter-rater reliability given that different translators may select different synonyms as the best equivalent for any given word. Moreover, nonprofessional translators with a limited vocabulary may use a word that does not exactly convey the intended message of the speaker. Despite assuming that the Azeri vocabulary of the study interpreters was smaller than their Persian vocabulary as discussed earlier, no significant differences were observed in mistranslation errors between Persian to Azeri and Azeri to Persian translation. The wider vocabulary of the raters, the majority of whom were professional translators, must have therefore caused inter-rater disagreements observed over mistranslation errors; nevertheless, given that the raters were asked to only count the errors, they cannot be compared, and this hypothesis cannot be approved.

According to the consecutive approach, the interpreter usually uses a “thought for thought” approach to understand a message and convey it in his own words, which are not necessarily the exact translation of the speaker’s words. Forgetting a phrase or sentence is the main disadvantage of this method. If the interpreter is not aware of forgetting a phrase, an omission error will occur; otherwise addition error will occur when he adds another phrase to his translation in a confabulation-like process. Compared to professional simultaneous interpreters, nonprofessional interpreters are more likely to omit or add a phrase. The present study found addition errors to be significantly more than omission errors, suggesting that the interpreters had been aware of their forgetfulness and made efforts to compensate for their memory errors. The interpreters living with the patients made more errors in both Persian to Azeri and Azeri to Persian translations.

Flores et al. showed that omission is the most frequent type of interpretation error, whereas the present research found (10)(10)(10) addition error to be the most frequent (10). This discrepancy of results can be explained by using the patients’ relatives as interpreters in the present study. The interpreters of Persian to Azeri translation who were not living with the patients made no addition or omission errors, suggesting that they could remember Persian better than Azeri. In this group, all omission errors were made by the relatives with lower education levels, while addition errors were made by the more educated patients.

The interpreters of Azeri to Persian translation who were living with the patients made fewer omission errors, while all of them made addition errors in their interpretation. In addition, the interpreters with lower education levels made more frequent omission and addition mistakes. As discussed before, their Persian vocabulary was directly related to their level of education, and they were quite likely to totally omit or replace a word or phrase for which they could not propose an appropriate equivalent in Persian.

More educated interpreters were therefore found to be more aware of their errors and to try to compensate for them by adding more details into their translation. Given their awareness of the patients’ conditions and medical statuses, the interpreters living with the patients were more likely to add something to their translation.

According to the raters, approximately 50% of all the errors were identified as “important” with potentially-negative effects on diagnosis or therapy, which is consistent with the figure (63%) reported in a study by Flores et al., although they found in another study that the proportion of errors with potential consequences for ad-hoc interpreters is 22%, which is slightly higher than that associated with no interpreters (20%). This discrepancy of results can be explained by the difference in the composition of ad-hoc interpreters in these studies. The majority of the errors labelled “important” in the present study were associated with mistranslation, which highlights the importance of using a professional interpreter in medical interviews, as was the case in previously-conducted studies (10–12). In case of unavailability of professional interpreters in the hospital, a bilingual interpreter, whose native language is the same as the patient’s, is recommended to be asked for cooperation. In case the patients’ relatives are the only alternative as bilingual interpreters, those who do not live the patients are preferred so as to reduce addition errors.

### Limitation

The present study was conducted in the emergency department of two referral hospitals, and the patients’ condition was more complicated compared to in community hospitals. Given that obtaining patient history is more complicated and detailed in a complex condition compared to in a simple atmosphere, the physicians might have asked more questions, making the interpreters more vulnerable to making mistakes.

Bilingual physicians were selected as the study raters. Professional translators are recommended to be selected and their ratings to be compared with those provided by these bilingual physicians in terms of error identification. Given the inability of professional interpreters to rate the clinical importance of the errors, these mistakes are recommended to be presented to other physicians to provide their comments.

Given that the raters were not supposed to record the exact word/phrase of the errors, providing examples of the errors is impossible in the present research.

## Conclusions

Language barrier negatively affects the patient-physician communication. Non-professional interpreters cannot correctly facilitate this communication, and their translation is prone to errors involving addition, omission and mistranslation, especially when translating from their native language to an official language. These errors can cause significant clinical consequences.
